# Sums of finite products of Chebyshev polynomials of the second kind and of Fibonacci polynomials

**DOI:** 10.1186/s13660-018-1744-5

**Published:** 2018-06-27

**Authors:** Taekyun Kim, Dae San Kim, Dmitry V. Dolgy, Jin-Woo Park

**Affiliations:** 1 Department of Mathematics, College of Science, Tianjin Polytechnic University, Tianjin, China; 2 Department of Mathematics, Kwangwoon University, Seoul, Republic of Korea; 3 Department of Mathematics, Sogang University, Seoul, Republic of Korea; 4 Hanrimwon, Kwangwoon University, Seoul, Republic of Korea; 5 Department of Mathematics Education, Daegu University, Gyeongsan-si, Republic of Korea

**Keywords:** 11B68, 11B83, 42A16, Fourier series, Chebyshev polynomials of the second kind, Fibonacci polynomials

## Abstract

In this paper, we consider sums of finite products of Chebyshev polynomials of the second kind and of Fibonacci polynomials and derive Fourier series expansions of functions associated with them. From these Fourier series expansions, we can express those sums of finite products in terms of Bernoulli polynomials and obtain some identities by using those expressions.

## Introduction and preliminaries

The Chebyshev polynomials $T_{n}(x)$ of the first kind, the Chebyshev polynomials $U_{n}(x)$ of the second kind, and the Fibonacci polynomials $F_{n}(x)$ are respectively defined by the recurrence relations as follows (see [13–15]):

1.1 $$\begin{aligned}& T_{n+2}(x)=2xT_{n+1}(x)-T_{n}(x)\quad (n\geq0), \qquad T_{0}(x)=1,\qquad T_{1}(x)=x, \end{aligned}$$

1.2 $$\begin{aligned}& U_{n+2}(x)=2xU_{n+1}(x)-U_{n}(x)\quad (n\geq0), \qquad U_{0}(x)=1,\qquad U_{1}(x)=2x, \end{aligned}$$

1.3 $$\begin{aligned}& F_{n+2}(x)=xF_{n+1}(x)+F_{n}(x)\quad (n\geq0),\qquad F_{0}(x)=0,\qquad F_{1}(x)=1. \end{aligned}$$

When $x=1$, $F_{n}=F_{n}(1)$ ($n\geq0$) is the Fibonacci sequence.

From (1.1), (1.2), and (1.3), it can be easily shown that the generating functions for $T_{n}(x)$, $U_{n}(x)$, and $F_{n}(x)$ are respectively given by (see [13–15]):

1.4 $$\begin{aligned}& \frac{1-xt}{1-2xt+t^{2}}=\sum_{n=0} ^{\infty} T_{n}(x)t^{n}, \end{aligned}$$

1.5 $$\begin{aligned}& F(t,x)=\frac{1}{1-2xt+t^{2}}=\sum_{n=0} ^{\infty}U_{n} (x)t^{n}, \end{aligned}$$

1.6 $$\begin{aligned}& G(t,x)=\frac{1}{1-xt-t^{2}}=\sum_{n=0} ^{\infty}F_{n+1}(x)t^{n}. \end{aligned}$$

As is well known, the *Bernoulli polynomials* $B_{n}(x)$ are defined by the generating function

1.7 $$ \frac{t}{e^{t}-1}e^{xt}=\sum _{n=0} ^{\infty}B_{n}(x)\frac{t^{n}}{n!}. $$

For any real number *x*, we let

1.8 $$ \langle x\rangle =x- [x ]\in [0, 1 ) $$

denote the fractional part of *x*, where $[x]$ indicates the greatest integer ≤*x*.

For any integers *m*, *r* with $m,r\geq1$, we put

1.9 $$ \alpha_{m,r}(x)=\sum_{i_{1}+i_{2}+\cdots+i_{r+1}=m} U_{i_{1}}(x)U_{i_{2}}(x)\cdots U_{i_{r+1}}(x), $$

where the sum runs over all nonnegative integers $i_{1},i_{2},\ldots ,i_{r+1}$ with $i_{1}+i_{2}+\cdots+i_{r+1}=m$.

Then we will consider the function $\alpha_{m,r}(\langle x\rangle)$ and derive their Fourier series expansions. As a corollary to these Fourier series expansions, we will be able to express $\alpha_{m,r}(x)$ in terms of Bernoulli polynomials $B_{n}(x)$. Indeed, our result here is as follows.

### Theorem A

*For any integers* *m*, *r* *with* $m,r\geq1$, *we let*

$$ \Delta_{m,r}=\frac{1}{2^{r}r!}\sum_{k=0} ^{ [\frac{m-1}{2} ]}(-1)^{k}\binom{m+r-k}{k}(m+r-2k)_{r}2^{m+r-2k}. $$

*Then we have the identity*

1.10 $$\begin{aligned} &\sum_{i_{1}+i_{2}+\cdots+i_{r+1}=m}U_{i_{1}}(x)U_{i_{2}}(x) \cdots U_{i_{r+1}}(x) \\ &\quad =\frac{1}{2r}\sum_{j=0} ^{m} 2^{j}\binom{r+j-1}{r-1}\Delta_{m-j+1,r+j-1}B_{j}(x). \end{aligned}$$

*Here* $(x)_{r}=x(x-1)\cdots(x-r+1)$ *for* $r\geq1$, *and* $(x)_{0}=1$.

*Also*, *for any integers* *m*, *r* *with* $m\geq1$, $r\geq2$, *we let*

1.11 $$ \beta_{m,r}(x)=\sum_{i_{1}+i_{2}+\cdots +i_{r}=m}F_{i_{1}+1}(x)F_{i_{2}+1}(x) \cdots F_{i_{r}+1}(x), $$

*where the sum runs over all nonnegative integers* $i_{1},i_{2},\ldots,i_{r}$ *with* $i_{1}+i_{2}+\cdots+i_{r}=m$.

Then we will consider the function $\beta_{m,r}(\langle x\rangle)$ and derive their Fourier series expansions. Again, as an immediate corollary to these, we can express $\beta_{m,r}(x)$ as a linear combination of Bernoulli polynomials. In detail, our result is as follows.

### Theorem B

*For any integers* *m*, *r* *with* $m\geq1$, $r\geq2$, *we let*

$$ \Omega_{m,r}=\sum_{l=0} ^{ [\frac{m-1}{2} ]} \binom {m+r-1-l}{l}\binom{m+r-1-2l}{r-1}. $$

*Then we have the identity*

1.12 $$\begin{aligned} &\sum_{i_{1}+i_{2}+\cdots+i_{r}=m}F_{i_{1}+1}(x)F_{i_{2}+1}(x) \cdots F_{i_{r}+1}(x) \\ &\quad =\frac{1}{r-1}\sum_{j=0} ^{m} \binom{r-2+j}{j}\Omega_{m-j+1,r+j-1}B_{j}(x). \end{aligned}$$

One particular thing we have to note here is that neither $U_{n}(x)$ nor $F_{n}(x)$ is Appell polynomials, while all our related results so far have been only about Appell polynomials (see [1, 5–8]).

Moreover, we will get some interesting identities that follow from Theorems A and B together with Lemmas 1 and 2 in [9].

As was mentioned in [7], studying these kinds of sums of finite products of special polynomials can be well justified by the following. Let us put

1.13 $$ \gamma_{m}(x)=\sum_{k=1} ^{m-1}\frac{1}{k(m-k)}B_{k}(x)B_{m-k}(x)\quad (m \geq2). $$

Then from the Fourier series expansion of $\gamma_{m}(\langle x\rangle)$ we can express $\gamma_{m}(x)$ in terms of Bernoulli polynomials just as in (1.10) and (1.12). Then, after some simple modification of this expression, we are able to obtain the famous Faber–Pandharipande–Zagier identity (see [3]) and some slightly different variant of Miki’s identity (see [2, 4, 10, 12]). For the details on this, the reader is referred to Introduction of the paper [7]. For some related results, we let the reader refer to the papers [1, 5–8].

## Fourier series expansions for functions associated with Chebyshev polynomials of the second kind

By differentiating equation (1.5) it was shown in [15] and mentioned in [13] that the sum of products in (1.9) can be neatly expressed as in the following. This will play a crucial role in this paper.

### Lemma 2.1

*Let* *n*, *r* *be nonnegative integers*. *Then we have the identity*

2.1 $$ \sum_{i_{1}+i_{2}+\cdots+i_{r+1}=n}U_{i_{1}}(x)U_{i_{2}}(x) \cdots U_{i_{r+1}}(x)=\frac{1}{2^{r}r!}U_{n+r} ^{(r)}(x), $$

*where the sum runs over all nonnegative integers* $i_{1},i_{2},\ldots ,i_{r+1}$ *with* $i_{1}+i_{2}+\cdots+i_{r+1}=n$.

It is well known that the Chebyshev polynomials of the second kind $U_{n}(x)$ are explicitly given by (see [11, 13])

2.2 $$ U_{n}(x)=\sum_{k=0} ^{ [\frac{n}{2} ]}(-1)^{k}\binom {n-k}{k}(2x)^{n-2k}. $$

The *r*th derivative of (2.1) is given by

2.3 $$ U_{n} ^{(r)}(x)=\sum _{k=0} ^{ [\frac{n-r}{2} ]}(-1)^{k}\binom {n-k}{k}(n-2k)_{r}2^{n-2k}x^{n-2k-r}. $$

Then, combining (2.1) and (2.3), we obtain

2.4 $$\begin{aligned} &\sum_{i_{1}+i_{2}+\cdots+i_{r+1}=n}U_{i_{1}}(x)U_{i_{2}}(x) \cdots U_{i_{r+1}}(x) \\ &\quad =\frac{1}{2^{r}r!}\sum_{k=0} ^{ [\frac{n}{2} ]}(-1)^{k}\binom{n+r-k}{k}(n+r-2k)_{r}2^{n+r-2k}x^{n-2k}. \end{aligned}$$

As in (1.9), we let

$$ \alpha_{m,r}(x)=\sum_{i_{1}+i_{2}+\cdots +i_{r+1}=m}U_{i_{1}}(x)U_{i_{2}}(x) \cdots U_{i_{r+1}}(x), $$

where $m,r\geq1$, and the sum runs over all nonnegative integers $i_{1},i_{2},\ldots,i_{r+1}$ with $i_{1}+i_{2}+\cdots+i_{r+1}=m$.

Then we will consider the function

2.5 $$ \alpha_{m,r}\bigl(\langle x\rangle\bigr)=\sum _{i_{1}+i_{2}+\cdots +i_{r+1}=m}U_{i_{1}}\bigl(\langle x\rangle \bigr)U_{i_{2}}\bigl(\langle x\rangle\bigr)\cdots U_{i_{r+1}}\bigl( \langle x\rangle\bigr), $$

defined on ${\mathbb{R}}$, which is periodic with period 1.

The Fourier series of $\alpha_{m,r}(\langle x\rangle)$ is

2.6 $$ \sum_{n=-\infty} ^{\infty}A_{n} ^{(m,r)}e^{2\pi inx}, $$

where

2.7 $$\begin{aligned} A_{n} ^{(m,r)}&= \int_{0} ^{1} \alpha_{m,r}\bigl(\langle x \rangle\bigr)e^{-2\pi inx}\,dx \\ &= \int_{0} ^{1} \alpha_{m,r}(x)e^{-2\pi inx} \,dx. \end{aligned}$$

For $m,r\geq1$, we put

2.8 $$\begin{aligned} \Delta_{m,r}&=\alpha_{m,r}(1)-\alpha_{m,r}(0) \\ &=\sum_{i_{1}+i_{2}+\cdots+i_{r+1}=m} \bigl(U_{i_{1}}(1)\cdots U_{i_{r+1}}(1)-U_{i_{1}}(0)\cdots U_{i_{r+1}}(0) \bigr). \end{aligned}$$

Then, for (2.4) and (2.8), we get

2.9 $$ \Delta_{m,r}=\frac{1}{2^{r}r!}\sum _{k=0} ^{ [\frac{m-1}{2} ]}(-1)^{k}\binom{m+r-k}{k}(m+r-2k)_{r}2^{m+r-2k}, $$

where we note that

2.10 $$ \alpha_{m,r}(0)= \textstyle\begin{cases} 0,&\text{if }m\text{ is odd}, \\ (-1)^{\frac{m}{2}}\binom{\frac{m}{2}+r}{\frac{m}{2}},&\text{if } m\text{ is even}. \end{cases} $$

Now, using (2.1), we note the following:

$$\begin{aligned} \frac{d}{dx}\alpha_{m,r}(x)&=\frac{d}{dx} \biggl( \frac {1}{2^{r}r!}U_{m+r} ^{(r)}(x) \biggr) \\ &=\frac{1}{2^{r}r!}U_{m+r} ^{(r+1)}(x) \\ &=2(r+1)\alpha_{m-1,r+1}(x). \end{aligned}$$

Thus we have shown that

2.11 $$ \frac{d}{dx}\alpha_{m,r}(x)=2(r+1) \alpha_{m-1,r+1}(x). $$

Replacing *m* by $m+1$ and *r* by $r-1$, from (2.11) we have

2.12 $$\begin{aligned}& \frac{d}{dx} \biggl(\frac{\alpha_{m+1,r-1}(x)}{2r} \biggr)= \alpha_{m,r}(x), \end{aligned}$$

2.13 $$\begin{aligned}& \int_{0} ^{1} \alpha_{m,r}(x)\,dx= \frac{1}{2r}\Delta_{m+1,r-1}, \end{aligned}$$

and

2.14 $$ \alpha_{m,r}(0)=\alpha_{m,r}(1)\quad \Longleftrightarrow\quad \Delta_{m,r}=0. $$

We are now ready to determine the Fourier coefficients $A_{n} ^{(m)}$.

*Case* 1: $n\neq0$.

2.15 $$\begin{aligned} A_{n} ^{(m,r)} =& \int_{0} ^{1} \alpha_{m,r}(x)e^{-2\pi inx} \,dx \\ =&-\frac{1}{2\pi in} \bigl[\alpha_{m,r}(x)e^{-2\pi inx} \bigr]_{0} ^{1} +\frac{1}{2\pi in} \int_{0} ^{1} \biggl(\frac{d}{dx}\alpha _{m,r}(x) \biggr)e^{-2\pi inx}\,dx \\ =&\frac{2(r+1)}{2\pi in} \int_{0} ^{1}\alpha_{m-1,r+1}(x)e^{-2\pi inx} \,dx-\frac{1}{2\pi in} \bigl(\alpha_{m,r}(1)-\alpha_{m,r}(0) \bigr) \\ =&\frac{2(r+1)}{2\pi in}A_{n} ^{(m-1,r+1)}-\frac{1}{2\pi in}\Delta _{m,r} \\ =&\frac{2(r+1)}{2\pi in} \biggl(\frac{2(r+2)}{2\pi in}A_{n} ^{(m-2,r+2)}- \frac{1}{2\pi in}\Delta_{m-1,r+1} \biggr)-\frac{1}{2\pi in} \Delta_{m,r} \\ =&\frac{2^{2}(r+2)_{2}}{(2\pi in)^{2}}A_{n} ^{(m-2,r+2)}-\sum _{j=1} ^{2}\frac {2^{j-1}(r+j-1)_{j-1}}{(2\pi in)^{j}}\Delta_{m-j+1,r+j-1} \\ =&\cdots \\ =&\frac{2^{m}(r+m)_{m}}{(2\pi in)^{m}}A_{n} ^{(0,r+m)}-\sum _{j=1} ^{m}\frac {2^{j-1}(r+j-1)_{j-1}}{(2\pi in)^{j}}\Delta_{m-j+1,r+j-1} \\ =&-\sum_{j=1} ^{m}\frac{2^{j-1}(r+j-1)_{j-1}}{(2\pi in)^{j}}\Delta _{m-j+1,r+j-1} \\ =&-\frac{1}{2r}\sum_{j=1} ^{m} \frac{2^{j}(r+j-1)_{j}}{(2\pi in)^{j}}\Delta _{m-j+1,r+j-1}. \end{aligned}$$

*Case* 2: $n=0$.

2.16 $$ A_{0} ^{(m,r)}= \int_{0} ^{1}\alpha_{m,r} (x)\,dx= \frac{1}{2r}\Delta_{m+1,r-1}. $$

Before proceeding further, we recall here that

- for $m\geq2$,
  2.17 $$ B_{m}\bigl(\langle x\rangle\bigr)=-m!\sum _{\substack{n=-\infty\\n\neq0}} ^{\infty}\frac {e^{2\pi inx}}{(2\pi in)^{m}}, $$
- for $m=1$,
  2.18 $$ -\sum_{\substack{n=-\infty\\n\neq0}} ^{\infty} \frac{e^{2\pi inx}}{2\pi in}= \textstyle\begin{cases} B_{1}(\langle x\rangle)&\text{for }x\in{\mathbb{R}}-{\mathbb{Z}}, \\ 0&\text{for }x\in{\mathbb{Z}}. \end{cases} $$

From (2.15)–(2.18), we now obtain the Fourier series of $\alpha_{m,r}(\langle x\rangle)$ given by

2.19 $$\begin{aligned} &\frac{1}{2r}\Delta_{m+1,r-1}-\sum_{\substack{n=-\infty\\n\neq0}} ^{\infty} \Biggl(\frac{1}{2r}\sum_{j=1} ^{m} \frac{2^{j}(r+j-1)_{j}}{(2\pi in)^{j}}\Delta_{m-j+1,r+j-1} \Biggr)e^{2\pi inx} \\ &\quad =\frac{1}{2r}\Delta_{m+1,r-1}+\frac{1}{2r}\sum _{j=1} ^{m} 2^{j}\binom {r+j-1}{r-1} \Delta_{m-j+1,r+j-1}\times \Biggl(-j!\sum_{\substack {n=-\infty\\n\neq0}} ^{\infty}\frac{e^{2\pi inx}}{(2\pi in)^{j}} \Biggr) \\ &\quad =\frac{1}{2r}\Delta_{m+1,r-1}+\frac{1}{2r}\sum _{j=2} ^{m} 2^{j} \binom {r+j-1}{r-1} \Delta_{m-j+1,r+j-1} \\ &\qquad {}+\Delta_{m,r}\times \textstyle\begin{cases} B_{1}(\langle x\rangle)&\text{for }x\in{\mathbb{R}}-{\mathbb{Z}}, \\ 0&\text{for } x\in{\mathbb{Z}}. \end{cases}\displaystyle \\ &\quad =\frac{1}{2r}\sum_{\substack{j=0\\j\neq1}} ^{m} 2^{j} \binom {r+j-1}{r-1}\Delta_{m-j+1,r+j-1}B_{j}\bigl( \langle x\rangle\bigr) \\ &\qquad {}+\Delta_{m,r}\times \textstyle\begin{cases} B_{1}(\langle x\rangle)&\text{for }x\in{\mathbb{R}}-{\mathbb{Z}}, \\ 0&\text{for }x\in{\mathbb{Z}}. \end{cases}\displaystyle \end{aligned}$$

$\alpha_{m,r}(\langle x\rangle)$ ($m,r\geq1$) is piecewise $C^{\infty}$. Moreover, $\alpha_{m,r}(\langle x\rangle)$ is continuous for those positive integers *m*, *r* with $\Delta_{m,r}=0$, and discontinuous with jump discontinuities at integers for those positive integers *m*, *r* with $\Delta_{m,r}\neq0$. Thus, for $\Delta_{m,r}=0$, the Fourier series of $\alpha_{m,r}(\langle x\rangle)$ converges uniformly to $\alpha_{m,r}(\langle x\rangle)$. On the other hand, for $\Delta_{m,r}\neq0$, the Fourier series of $\alpha_{m,r}(\langle x\rangle)$ converges pointwise to $\alpha_{m,r}(\langle x\rangle)$ for $x\in{\mathbb{R}}-{\mathbb{Z}}$, and converges to

2.20 $$ \frac{1}{2} \bigl(\alpha_{m,r}(0)+ \alpha_{m,r}(1) \bigr)=\alpha _{m,r}(0)+\frac{1}{2} \Delta_{m,r} $$

for $x\in{\mathbb{Z}}$.

From these observations together with (2.19) and (2.20), we have the next two theorems.

### Theorem 2.2

*For any integers* *m*, *r* *with* $m,r\geq1$, *we let*

$$ \Delta_{m,r}=\frac{1}{2^{r}r!}\sum_{k=0} ^{ [\frac{m-1}{2} ]}(-1)^{k}\binom{m+r-k}{k}(m+r-2k)_{r}2^{m+r-2k}. $$

*Assume that* $\Delta_{m,r}=0$ *for some positive integers* $m,r$. *Then we have the following*:

- $$ \sum_{i_{1}+i_{2}+\cdots+i_{r+1}=m}U_{i_{1}}\bigl(\langle x\rangle \bigr)U_{i_{2}}\bigl(\langle x\rangle\bigr)\cdots U_{i_{r+1}}\bigl( \langle x\rangle\bigr) $$
  *has the Fourier series expansion*
  $$\begin{aligned} &\sum_{i_{1}+i_{2}+\cdots+i_{r+1}=m}U_{i_{1}}\bigl(\langle x\rangle \bigr)U_{i_{2}}\bigl(\langle x\rangle\bigr)\cdots U_{i_{r+1}}\bigl( \langle x\rangle\bigr) \\ &\quad =\frac{1}{2r}\Delta_{m+1,r-1}-\sum _{\substack{n=-\infty\\n\neq 0}} ^{\infty} \Biggl(\frac{1}{2r}\sum _{j=1} ^{m} \frac {2^{j}(r+j-1)_{j}}{(2\pi in)^{j}}\Delta_{m-j+1,r+j-1} \Biggr)e^{2\pi inx} \end{aligned}$$
  *for all* $x\in{\mathbb{R}}$, *where the convergence is uniform*.
- $$\begin{aligned} &\sum_{i_{1}+i_{2}+\cdots+i_{r+1}=m}U_{i_{1}}\bigl(\langle x\rangle \bigr)U_{i_{2}}\bigl(\langle x\rangle\bigr)\cdots U_{i_{r+1}}\bigl( \langle x\rangle\bigr) \\ &\quad =\frac{1}{2r}\sum_{\substack{j=0\\j\neq1}} ^{m} 2^{j}\binom {r+j-1}{r-1}\Delta_{m-j+1,r+j-1}B_{j}\bigl( \langle x\rangle\bigr) \end{aligned}$$
  *for all* $x\in{\mathbb{R}}$.

### Theorem 2.3

*For any integers* *m*, *r* *with* $m,r\geq1$, *we let*

$$ \Delta_{m,r}=\frac{1}{2^{r}r!}\sum_{k=0} ^{ [\frac{m-1}{2} ]}(-1)^{k}\binom{m+r-k}{k}(m+r-2k)_{r}2^{m+r-2k}. $$

*Assume that* $\Delta_{m,r}\neq0$ *for some positive integers* *m*, *r*. *Then we have the following*:

- $$\begin{aligned} &\frac{1}{2r}\Delta_{m+1,r-1}-\sum_{\substack{n=-\infty\\n \neq 0}} ^{\infty} \Biggl(\frac{1}{2r}\sum_{j=1} ^{m} \frac {2^{j}(r+j-1)_{j}}{(2\pi in)^{j}}\Delta_{m-j+1,r+j-1} \Biggr)e^{2\pi inx} \\ &\quad = \textstyle\begin{cases} \sum_{i_{1}+i_{2}+\cdots+i_{r+1}=m}U_{i_{1}}(\langle x\rangle)U_{i_{2}}(\langle x\rangle)\cdots U_{i_{r+1}}(\langle x\rangle)&\textit{for }x\in\mathbb{R}-\mathbb{Z}, \\ \frac{1}{2}\Delta_{m,r}& \textit{for }x\in{\mathbb{Z}} \textit{ and }m \textit{ odd}, \\ (-1)^{\frac{m}{2}}\binom{\frac{m}{2}+r}{\frac{m}{2}}+\frac {1}{2}\Delta_{m,r} &\textit{for }x\in{\mathbb{Z}}\textit{ and }m\textit{ even}. \end{cases}\displaystyle \end{aligned}$$
- $$\begin{aligned} &\frac{1}{2r}\sum_{j=0} ^{m} 2^{j} \binom{r+j-1}{r-1}\Delta _{m-j+1,r+j-1}B_{j}\bigl( \langle x\rangle\bigr) \\ &\quad =\sum_{i_{1}+i_{2}+\cdots+i_{r+1}=m}U_{i_{1}}\bigl(\langle x \rangle\bigr)U_{i_{2}}\bigl(\langle x\rangle\bigr)\cdots U_{i_{r+1}} \bigl(\langle x\rangle\bigr)\quad \textit{for }x\in{\mathbb{R}}-{\mathbb{Z}}; \\ &\frac{1}{2r}\sum_{\substack{j=0\\j\neq1}} ^{m} 2^{j} \binom {r+j-1}{r-1}\Delta_{m-j+1,r+j-1}B_{j}\bigl( \langle x\rangle\bigr) \\ &\quad = \textstyle\begin{cases} \frac{1}{2}\Delta_{m,r}&\textit{for }x\in\mathbb{Z}\textit{ and }m \textit{ odd}, \\ (-1)^{\frac{m}{2}}\binom{\frac{m}{2}+r}{\frac{m}{2}}+\frac {1}{2}\Delta_{m,r}&\textit{for }x\in{\mathbb{Z}}\textit{ and }m\textit{ even}. \end{cases}\displaystyle \end{aligned}$$

From Theorems 2.2 and 2.3, we immediately obtain the stated result in Theorem A expressing $\alpha_{m,r}(x)$ as a linear combination of Bernoulli polynomials.

## Fourier series expansions for functions associated with Fibonacci polynomials

The following lemma is stated as equation (7) in [14] which is important for our purpose.

### Lemma 3.1

*Let* *n*, *r* *be integers with* $n\geq0$, $r\geq1$. *Then we have the identity*

3.1 $$ \sum_{i_{1}+i_{2}+\cdots+i_{r}=n}F_{i_{1}+1}(x)F_{i_{2}+1}(x) \cdots F_{i_{r}+1}(x)=\frac{1}{(r-1)!}F_{n+r} ^{(r-1)}(x), $$

*where the sum runs over all nonnegative integers* $i_{1},i_{2},\ldots,i_{r}$ *with* $i_{1}+i_{2}+\cdots+i_{r}=n$.

An explicit expression for $F_{n+1}(x)$ ($n\geq0$) is stated in equation (9) of [14].

3.2 $$ F_{n+1}(x)=\sum_{l=0} ^{ [\frac{n}{2} ]}\binom{n-l}{l}x^{n-2l}. $$

As was noted in (10) of [14], the $(r-1)$th derivative of $F_{n+r}(x)$ is

3.3 $$ F_{n+r} ^{(r-1)}(x)=\sum _{l=0} ^{ [\frac{n}{2} ]}\frac {(n+r-1-l)!}{l!(n-2l)!}x^{n-2l}. $$

In addition, it was also noted in [14] that, combining (3.1) and (3.3), we have

3.4 $$\begin{aligned} &\sum_{i_{1}+i_{2}+\cdots+i_{r}=n}F_{i_{1}+1}(x)F_{i_{2}+1}(x) \cdots F_{i_{r}+1}(x) \\ &\quad =\sum_{l=0} ^{ [\frac{n}{2} ]}\binom{n+r-1-l}{l} \binom {n+r-1-2l}{r-1}x^{n-2l}. \end{aligned}$$

As in (1.11), we let

$$ \beta_{m,r}(x)=\sum_{i_{1}+i_{2}+\cdots +i_{r}=m}F_{i_{1}+1}(x)F_{i_{2}+1}(x) \cdots F_{i_{r}+1}(x), $$

where $m\geq1$, $r\geq2$, and the sum runs over all nonnegative integers $i_{1},i_{2},\ldots,i_{r}$ with $i_{1}+i_{2}+\cdots+i_{r}=m$.

Then we will consider the function

3.5 $$ \beta_{m,r}\bigl(\langle x\rangle\bigr)=\sum _{i_{1}+i_{2}+\cdots +i_{r}=m}F_{i_{1}+1}\bigl(\langle x\rangle \bigr)F_{i_{2}+1}\bigl(\langle x\rangle\bigr)\cdots F_{i_{r}+1}\bigl( \langle x\rangle\bigr), $$

defined on ${\mathbb{R}}$, which is periodic with period 1.

The Fourier series of $\beta_{m,r}(\langle x\rangle)$ is

3.6 $$ \sum_{n=-\infty} ^{\infty} B_{n} ^{(m,r)}e^{2\pi inx}, $$

where

3.7 $$\begin{aligned} B_{n} ^{(m,r)}&= \int_{0} ^{1}\beta_{m,r}\bigl(\langle x \rangle\bigr)e^{-2\pi inx}\,dx \\ &= \int_{0} ^{1} \beta_{m,r}(x)e^{-2\pi inx} \,dx. \end{aligned}$$

For $m\geq1$, $r\geq2$, we set

3.8 $$\begin{aligned} \Omega_{m,r}&=\beta_{m,r}(1)-\beta_{m,r}(0) \\ &=\sum_{i_{1}+i_{2}+\cdots+i_{r}=m} \bigl(F_{i_{1}+1}(1)\cdots F_{i_{r}+1}(1)-F_{i_{1}+1}(0)\cdots F_{i_{r}+1}(0) \bigr). \end{aligned}$$

Then, from (3.4) and (3.8), we have

3.9 $$ \Omega_{m,r}=\sum_{l=0} ^{ [\frac{m-1}{2} ]}\binom {m+r-1-l}{l}\binom{m+r-1-2l}{r-1}. $$

In particular, we note that $\Omega_{m,r}>0$ for any $m\geq1$, $r\geq 2$. Also, we note that

3.10 $$ \beta_{m,r}(0)= \textstyle\begin{cases} 0,&\text{if }m\mbox{ is odd}, \\ \binom{\frac{m}{2}+r-1}{\frac{m}{2}},&\text{if }m\mbox{ is even}. \end{cases} $$

Now, using (3.1), we see the following:

$$\begin{aligned} \frac{d}{dx}\beta_{m,r}(x)&=\frac{d}{dx} \biggl( \frac {1}{(r-1)!}F_{m+r} ^{(r-1)}(x) \biggr) \\ &=\frac{1}{(r-1)!}F_{m+r} ^{(r)}(x) \\ &=r\beta_{m-1,r+1}(x). \end{aligned}$$

Thus we have shown that

3.11 $$ \frac{d}{dx}\beta_{m,r}(x)=r\beta_{m-1,r+1}(x). $$

Replacing *m* by $m+1$ and *r* by $r-1$, from (3.11) we get

3.12 $$\begin{aligned}& \frac{d}{dx} \biggl(\frac{\beta_{m+1,r-1}(x)}{r-1} \biggr)= \beta_{m,r}(x), \end{aligned}$$

3.13 $$\begin{aligned}& \int_{0} ^{1} \beta_{m,r}(x)\,dx= \frac{1}{r-1}\Omega_{m+1,r-1}, \end{aligned}$$

and

3.14 $$ \beta_{m,r}(0)=\beta_{m,r}(1) \quad \Longleftrightarrow\quad \Omega_{m,r}=0. $$

We are now going to determine the Fourier coefficients $B_{n} ^{(m)}$.

*Case* 1: $n\neq0$.

3.15 $$\begin{aligned} B_{n} ^{(m,r)}&= \int_{0} ^{1} \beta_{m,r}(x)e^{-2\pi inx} \,dx \\ &=-\frac{1}{2\pi in} \bigl[\beta_{m,r}(x)e^{-2\pi inx} \bigr]_{0} ^{1} +\frac{1}{2\pi in} \int_{0} ^{1} \biggl(\frac{d}{dx} \beta_{m,r}(x) \biggr)e^{-2\pi inx}\,dx \\ &=\frac{r}{2\pi in} \int_{0} ^{1}\beta_{m-1,r+1}(x)e^{-2\pi inx} \,dx-\frac {1}{2\pi in} \bigl(\beta_{m,r}(1)-\beta_{m,r}(0) \bigr) \\ &=\frac{r}{2\pi in}B_{n} ^{(m-1,r+1)}-\frac{1}{2\pi in} \Omega_{m,r} \\ &=\frac{r}{2\pi in} \biggl(\frac{r+1}{2\pi in}B_{n} ^{(m-2,r+2)}- \frac {1}{2\pi in}\Omega_{m-1,r+1} \biggr)-\frac{1}{2\pi in} \Omega_{m,r} \\ &=\frac{(r+1)_{2}}{(2\pi in)^{2}}B_{n} ^{(m-2,r+2)}-\sum _{j=1} ^{2}\frac {(r+j-2)_{j-1}}{(2\pi in)^{j}}\Omega_{m-j+1,r+j-1} \\ &=\cdots \\ &=\frac{(r+2)_{m}}{(2\pi in)^{m}}B_{n} ^{(0,r+m)}-\sum _{j=1} ^{m}\frac {(r+j-2)_{j-1}}{(2\pi in)^{j}}\Omega_{m-j+1,r+j-1} \\ &=-\sum_{j=1} ^{m}\frac{(r+j-2)_{j-1}}{(2\pi in)^{j}}\Omega _{m-j+1,r+j-1} =-\frac{1}{r-1}\sum_{j=1} ^{m} \frac{(r+j-2)_{j}}{(2\pi in)^{j}}\Omega _{m-j+1,r+j-1}. \end{aligned}$$

*Case* 2: $n=0$.

3.16 $$ B_{0} ^{(r,m)}= \int_{0} ^{1}\beta_{m,r}(x)\,dx= \frac{1}{r-1}\Omega_{m+1,r-1}. $$

From (2.17), (2.18), (3.15), and (3.16), we now have the following Fourier series expansion of $\beta_{m,r}(\langle x\rangle)$ given by

3.17 $$\begin{aligned} &\frac{1}{r-1}\Omega_{m+1,r-1}-\sum_{\substack{n=-\infty\\n\neq 0}} ^{\infty} \Biggl(\frac{1}{r-1}\sum_{j=1} ^{m}\frac {(r+j-2)_{j}}{(2\pi in)^{j}}\Omega_{m-j+1,r+j-1} \Biggr)e^{2\pi inx} \\ &\quad =\frac{1}{r-1}\Omega_{m+1,r-1} \\ &\qquad {}+\frac{1}{r-1}\sum_{j=1} ^{m} \binom{r-2+j}{j}\Omega _{m-j+1,r+j-1}\times \Biggl(-j!\sum _{\substack{n=-\infty\\n\neq0}} ^{\infty}\frac{e^{2\pi inx}}{(2\pi in)^{j}} \Biggr) \\ &\quad =\frac{1}{r-1}\Omega_{m+1,r-1}+\frac{1}{r-1}\sum _{j=2} ^{m} \binom {r-2+j}{j}\Omega_{m-j+1,r+j-1}B_{j} \bigl(\langle x\rangle\bigr) \\ &\qquad {}+\Omega_{m,r}\times \textstyle\begin{cases} B_{1} (\langle x\rangle)&\text{for }x\in{\mathbb{R}}-{\mathbb{Z}}, \\ 0&\text{for }x\in{\mathbb{Z}} \end{cases}\displaystyle \\ &\quad =\frac{1}{r-1}\sum_{\substack{j=0\\j\neq1}} ^{m} \binom {r-2+j}{j}\Omega_{m-j+1,r+j-1}B_{j}\bigl(\langle x\rangle\bigr) \\ &\qquad {}+\Omega_{m,r}\times \textstyle\begin{cases} B_{1}(\langle x\rangle)&\text{for }x\in{\mathbb{R}}-{\mathbb{Z}}, \\ 0&\text{for }x\in{\mathbb{Z}}. \end{cases}\displaystyle \end{aligned}$$

$\beta_{m,r}(\langle x\rangle)$ ($m\geq1$, $r\geq2$) is piecewise $C^{\infty}$ and discontinuous with jump discontinuities at integers, as $\Omega _{m,r}>0$ for any $m\geq1$, $r\geq2$. Thus the Fourier series of $\beta_{m,r}(\langle x\rangle)$ converges pointwise to $\beta_{m,r}(\langle x\rangle)$ for $x\in{\mathbb{R}}-{\mathbb{Z}}$, and converges to

3.18 $$ \frac{1}{2} \bigl(\beta_{m,r}(0)+ \beta_{m,r}(1) \bigr)=\beta _{m,r}(0)+\frac{1}{2} \Omega_{m,r} $$

for $x\in{\mathbb{Z}}$.

From these observations together with (3.17) and (3.18), we have the following theorem.

### Theorem 3.2

*For any integers* *m*, *r* *with* $m\geq1$, $r\geq2$, *we let*

$$ \Omega_{m,r}=\sum_{l=0} ^{ [\frac{m-1}{2} ]} \binom {m+r-1-l}{l}\binom{m+r-1-2l}{r-1}. $$

*Then we have the following*:

- $$\begin{aligned} &\frac{1}{r-1}\Omega_{m+1,r-1}-\sum_{\substack{n=-\infty\\n\neq 0}} ^{\infty} \Biggl(\frac{1}{r-1}\sum_{j=1} ^{m} \frac {(r-2+j)_{j}}{(2\pi in)^{j}}\Omega_{m-j+1,r+j-1} \Biggr)e^{2\pi inx} \\ &\quad = \textstyle\begin{cases} \sum_{i_{1}+i_{2}+\cdots+i_{r}=m}F_{i_{1}+1}(\langle x\rangle)F_{i_{2}+1}(\langle x\rangle)\cdots F_{i_{r}+1}(\langle x\rangle)&\textit{for }x\in{\mathbb{R}}-{\mathbb{Z}}, \\ \frac{1}{2}\Omega_{m,r}& \textit{for }x\in{\mathbb{Z}}\textit{ and }m\textit{ odd}, \\ \binom{\frac{m}{2}+r-1}{\frac{m}{2}}+\frac{1}{2}\Omega_{m,r}& \textit{for }x\in{\mathbb{Z}}\textit{ and }m\textit{ even}. \end{cases}\displaystyle \end{aligned}$$
- $$\begin{aligned} &\frac{1}{r-1}\sum_{j=0} ^{m} \binom{r-2+j}{j}\Omega _{m-j+1,r+j-1}B_{j}\bigl(\langle x\rangle \bigr) \\ &\quad =\sum_{i_{1}+i_{2}+\cdots+i_{r}=m}F_{i_{1}+1}\bigl(\langle x \rangle\bigr)F_{i_{2}+1}\bigl(\langle x\rangle\bigr)\cdots F_{i_{r}+1} \bigl(\langle x\rangle\bigr)\quad \textit{for }x\in{\mathbb{R}}-{\mathbb{Z}}; \\ &\frac{1}{r-1}\sum_{\substack{j=0\\j\neq1}} ^{m} \binom {r-2+j}{j}\Omega_{m-j+1,r+j-1}B_{j}\bigl(\langle x\rangle\bigr) \\ &\quad = \textstyle\begin{cases} \frac{1}{2}\Omega_{m,r}&\textit{for }x\in{\mathbb{Z}}\textit{ and }m \textit{ odd}, \\ \binom{\frac{m}{2}+r-1}{\frac{m}{2}}+\frac{1}{2}\Omega _{m,r}&\textit{for }x\in{\mathbb{Z}}\textit{ and }m\textit{ even}. \end{cases}\displaystyle \end{aligned}$$

From Theorem 3.2, we immediately get the result in Theorem B expressing $\beta_{m,r}(x)$ as a linear combination of Bernoulli polynomials.

## Applications

Let $T_{n}(x)$ ($n\geq0$) be the Chebyshev polynomials of the first kind given by (1.1) or (1.4). We need the following lemma from [9].

### Lemma 4.1

([9, Lemmas 1, 2])

*Let* $n\geq0$, $m\geq1$ *be integers*. *Then we have the following*:

4.1 $$\begin{aligned}& U_{n} \biggl(\frac{\sqrt{-1}}{2} \biggr)= (\sqrt{-1} )^{n}F_{n+1}, \end{aligned}$$

4.2 $$\begin{aligned}& U_{n} \biggl(T_{m} \biggl(\frac{\sqrt{-1}}{2} \biggr) \biggr)= (\sqrt {-1} )^{mn}\frac{F_{m(n+1)}}{F_{m}}, \end{aligned}$$

4.3 $$\begin{aligned}& U_{n} \bigl(T_{m}(x) \bigr)=\frac{U_{m(n+1)-1}(x)}{U_{m-1}(x)}. \end{aligned}$$

Substituting $x=\frac{\sqrt{-1}}{2}$ into (1.10) and using (4.1), we have

4.4 $$\begin{aligned} &(\sqrt{-1})^{m}\sum_{i_{1}+i_{2}+\cdots+i_{r+1}=m} F_{i_{1}+1}F_{i_{2}+1}\cdots F_{i_{r+1}+1} \\ &\quad =\frac{1}{2r}\sum_{j=0} ^{m} 2^{j}\binom{r+j-1}{r-1}\Delta _{m-j+1,r+j-1}B_{j} \biggl( \frac{\sqrt{-1}}{2} \biggr). \end{aligned}$$

On the other hand, with $x=1$ and replacing *r* by $r+1$, from (1.12) we obtain

4.5 $$\begin{aligned} &\sum_{i_{1}+i_{2}+\cdots+i_{r+1}=m} F_{i_{1}+1}F_{i_{2}+1}\cdots F_{i_{r+1}+1} \\ &\quad =\frac{1}{r}\sum_{j=0} ^{m} \binom{r-1+j}{j}\Omega_{m-j+1,r+j}B_{j}(1). \end{aligned}$$

Combining (4.4) and (4.5), we get

$$\begin{aligned} &\sum_{i_{1}+i_{2}+\cdots+i_{r+1}=m} F_{i_{1}+1}F_{i_{2}+1}\cdots F_{i_{r+1}+1} \\ &\quad =\frac{1}{2r}(-\sqrt{-1})^{m}\sum _{j=0} ^{m} 2^{j} \binom {r+j-1}{r-1} \Delta_{m-j+1,r+j-1}B_{j} \biggl(\frac{\sqrt{-1}}{2} \biggr) \\ &\quad =\frac{1}{r}\sum_{j=0} ^{m} \binom{r-1+j}{j}\Omega _{m-j+1,r+j}B_{j}+\Omega_{m,r+1}. \end{aligned}$$

Substituting $T_{a} (\frac{\sqrt{-1}}{2} )$ for *x* in (1.10) and using (4.2), we get: for any positive integer *a*,

$$\begin{aligned} &\sum_{i_{1}+i_{2}+\cdots+i_{r+1}=m}F_{a(i_{1}+1)}F_{a(i_{2}+1)}\cdots F_{a(i_{r+1}+1)} \\ &\quad =\frac{1}{2r}(-\sqrt{-1})^{am}F_{a} ^{r+1} \sum_{j=0} ^{m} 2^{j} \binom {r+j-1}{r-1}\Delta_{m-j+1,r+j-1}B_{j} \biggl(T_{a} \biggl(\frac{\sqrt {-1}}{2} \biggr) \biggr). \end{aligned}$$

Finally, replacing *x* by $T_{a}(x)$ in (1.10) and using (4.3), we have: for any positive integer *a*,

$$\begin{aligned} &\sum_{i_{1}+i_{2}+\cdots +i_{r+1}=m}U_{a(i_{1}+1)-1}(x)U_{a(i_{2}+1)-1}(x) \cdots U_{a(i_{r+1}+1)-1}(x) \\ &\quad =\frac{1}{2r} \bigl(U_{a-1} (x) \bigr)^{r+1}\sum _{j=1} ^{m} 2^{j} \binom{r+j-1}{r-1}\Delta_{m-j+1,r+j-1}B_{j} \bigl(T_{a}(x) \bigr). \end{aligned}$$

## Results and discussion

In this paper, we study sums of finite products of Chebyshev polynomials of the second kind and of Fibonacci polynomials and derive Fourier series expansions of functions associated with them. From these Fourier series expansions, we can express those sums of finite products in terms of Bernoulli polynomials and obtain some identities by using those expressions. The Fourier series expansion of the Chebyshev polynomials and Fibonacci polynomials are useful in computing the special values of zeta function or some special functions (see [5, 7, 9, 11, 13–15]). It is expected that the Fourier series of those polynomials will find some applications in relationship to the generalizations of the special zeta functions.

## Conclusion

In this paper, we considered the Fourier series expansions of functions associated with Chebyshev polynomials of the second kind and of Fibonacci polynomials. The Fourier series are determined completely.
